# Time-Resolved Photoluminescence Microscopy for the Analysis of Semiconductor-Based Paint Layers

**DOI:** 10.3390/ma10111335

**Published:** 2017-11-21

**Authors:** Daniela Comelli, Alessia Artesani, Austin Nevin, Sara Mosca, Victor Gonzalez, Myriam Eveno, Gianluca Valentini

**Affiliations:** 1 Physics Department, Politecnico di Milano, Piazza Leonardo da Vinci, 20133 Milano, Italy; alessia.artesani@polimi.it (A.A.); sara.mosca@polimi.it (S.M.); gianluca.valentini@polimi.it (G.V.); 2 Istituto di Fotonica e Nanotecnologie-Consiglio Nazionale delle Ricerche (IFN-CNR), Piazza Leonardo da Vinci, 20133 Milano, Italy; austinnevin@gmail.com; 3 Centre de Recherche et de Restauration des Musées de France (C2RMF), Palais du Louvre, F-75001 Paris, France; victor.gonzalez@culture.gouv.fr (V.G.); myriam.eveno@culture.gouv.fr (M.E.); 4 Chimie Paris-Tech, PSL Research University, CNRS, Institut de Recherche de Chimie Paris (IRCP), F-75005 Paris, France

**Keywords:** time-resolved photoluminescence, photoluminescence microscopy, semiconductor pigments, trap state levels, zinc white, cadmium yellow

## Abstract

In conservation, science semiconductors occur as the constituent matter of the so-called semiconductor pigments, produced following the Industrial Revolution and extensively used by modern painters. With recent research highlighting the occurrence of various degradation phenomena in semiconductor paints, it is clear that their detection by conventional optical fluorescence imaging and microscopy is limited by the complexity of historical painting materials. Here, we illustrate and prove the capabilities of time-resolved photoluminescence (TRPL) microscopy, equipped with both spectral and lifetime sensitivity at timescales ranging from nanoseconds to hundreds of microseconds, for the analysis of cross-sections of paint layers made of luminescent semiconductor pigments. The method is sensitive to heterogeneities within micro-samples and provides valuable information for the interpretation of the nature of the emissions in samples. A case study is presented on micro samples from a painting by Henri Matisse and serves to demonstrate how TRPL can be used to identify the semiconductor pigments zinc white and cadmium yellow, and to inform future investigations of the degradation of a cadmium yellow paint.

## 1. Introduction

Photoluminescence (PL) microscopy is a powerful optical method for the study of crystal defects in semiconductors and organometallic complexes [1], with important applications in the manufacturing process of nanostructures [2,3,4], optoelectronic devices and solar cell systems [5].

Semiconductors are the constituent matter of the so-called semiconductor pigments. Illustrative examples of this class of coloring matter include ancient pigments, such as the brilliant red vermillion (HgS), first used during the Neolithic Age [6], and the lemon yellow orpiment (As_2_S_3_), extensively found on ancient Egyptian objects and paintings. Thereafter, a variety of novel semiconductor pigments were produced in the Modern Age, starting from the second half of the 19th century when synthetic semiconductor pigments were introduced, and include zinc white (ZnO), zinc sulphide (ZnS), titanium white (TiO_2_), cadmium yellow (Zn_x_Cd_1−x_S) and red (CdS_1−x_Se_x_). These synthetic pigments were extensively used by painters, and offered new hues and significantly greater covering power than many paints based on natural minerals.

While much work has been carried out on the characterization of the photo-physical and optical properties of semiconductors, both in bulk materials and nanostructures, modern semiconductor pigments have received less attention [7,8,9,10,11]. Recent research has highlighted the complexity of historical semiconductor pigment samples, elucidating the presence of trace metal impurities [12] and various reaction products [13,14] following the imperfect synthesis processes developed at the beginning of the Second Industrial Revolution. The situation is further complicated in real painted layers, where the interaction of pigments with surrounding inorganic and organic compounds can give rise to various degradation phenomena induced by the formation of metal carboxylates [15,16] and by oxo-reduction processes [17,18].

In this context, analytical methods with micro- and nano-mapping capabilities become essential for probing the spatial heterogeneities of historical samples. The great interest in this kind of analyses is demonstrated by recent outstanding research on cultural heritage materials. Non-linear microscopy and nanoprobe FTIR have been employed for the visualization of fibrillar collagen in degraded parchments [19], while high-resolution nanoprobe X-ray fluorescence has been utilised for mapping metal impurities in submicron particles of zinc white paints [14]. A novel synchrotron-based full-field photoluminescence microscope has been effectively employed to probe the spatial fluctuation of crystal defects in historical zinc white paints [20], and of cuprous oxide phases in an ancient copper amulet [21].

Fluorescence lifetime imaging microscopy (FLIM) is a standard technique for probing tiny emission lifetime differences in microscopy samples [22], and both commercial and research-level devices with very high temporal (close to few picoseconds) [22] and spatial resolution are now available. High spatial resolution can also be achieved with advanced super-resolution microscopy methods [23]. FLIM is widely employed in many application fields, ranging from biology [24] to material science, including nanomaterials [25]. Recently, the method has been extended to the analysis of longer (microsecond) emission lifetimes. In this latter case, it is typically referred to as phosphorescence lifetime imaging microscopy (PLIM) [26] and it is employed for the sensing of cell environmental properties by probing the emission lifetimes of specific metal–ion complexes [26]. FLIM and PLIM set-ups are typically based on time-correlated-single-photon-counting (TCSPC) detectors and femtosecond mode-locking laser sources, while imaging of samples is achieved through sample raster scanning. However, these devices are designed for probing emission lifetimes in a specific time range, and are thus poorly suited for the analysis of heterogeneous samples with lifetimes ranging from nanosecond to hundreds of microseconds. In this latter case, the use of a time-gated approach [27] is preferred.

Paint stratigraphic microsamples are heterogeneous samples, typically made of different, and unknown, paint layers, with each layer being a mixture of pigments, binding media, additives and possible reaction products. As a consequence, the optical emission from these samples can be very heterogeneous, not only in terms of spatial distribution, but also in terms of decay kinetics and spectral properties. Therefore time-resolved imaging of such heterogeneities requires a versatile and flexible device, capable of measuring the PL emissions in various spectral regions, and the detection of both fast and slow emission decay kinetics. Recently, we have demonstrated the potential of lifetime-resolved and spectrally-resolved PL microscopies for the detection of luminescent centers, ascribed to metal substitutions, in historical samples of the lithopone pigment (ZnS + BaSO_4_) [12]. On the basis of our initial study, we have now developed a novel and optimized time-resolved PL (TRPL) microscope, based on a time-gated camera, which combines both multispectral and lifetime detection with a temporal resolution spanning from nanosecond to hundreds of microseconds. The system, characterized by a micrometric spatial resolution, allows the detection of emission lifetimes in a wide temporal range. This is a key feature for the in-depth investigation of heterogeneous semiconductor materials characterized by PL emissions occurring following both direct band-edge (BE) and trap state (TS) recombination paths.

In the present work, the advantages of such a novel setup are illustrated through the study of precious stratigraphic micro-samples from a painting by Henri Matisse (1869–1954), which present interesting questions on the use of modern pigments by the famous Post-Impressionist painter.

## 2. Materials and Methods

### 2.1. The Micro-Samples

Two micro-samples, originally taken in 1977 in different locations of Henri Matisse’s painting *Still Life with Eggplant* (Musée des Beaux-Arts, Grenoble, France, 210 × 245 cm, 1911, Figure S1 in Supplementary Materials), have been selected for the present research. Prior to analysis, samples were prepared as stratigraphic cross sections, by embedding them in polyester resin. For scanning electron microscopy-energy dispersive X-ray spectroscopy (SEM-EDX) analysis, the stratigraphic cross-sections were carbon coated. In the rest of this text, samples will be quoted with the same numbering used for their archiving at the Centre de Recherche et de Restauration des Musées de France (C2RMF) (2105 and 2107).

*Still Life with Eggplant,* painted in Collioure in 1911, belongs to the ensemble of Symphonic Interiors and is considered one of the most important paintings by Matisse preserved in France. Previous analysis on microsamples [28] have revealed that the painting was painted on an unprimed linen cloth and that animal glue has been used as pigment binder.

### 2.2. TRPL Microscopy

The system is based on a Q-switching laser source (FTSS 355-50, Crylas GmbH, Berlin, Germany, pulse energy = 70 μJ, pulse duration = 1.0 ns, repetition rate = 100 Hz), emitting light pulses at 355 nm, and on a time-gated camera, made of an image intensifier (C9546-03, Hamamatsu Photonics, Hamamatsu, Japan) optically coupled to a cooled monochrome digital camera (R6, Qimaging, Surrey, BC, Canada). The image intensifier is capable of gated detection with a temporal width adjustable from few nanoseconds to the continuous mode. In conservation science, this imaging device has been used extensively to capture the emission decay kinetics of a variety of artworks [29,30,31,32]. In this work both the laser and the time-gated image detector have been coupled to a microscopy stage and to a filter wheel, placed in the detection path, in order to analyze the decay kinetics and the spectral properties of the optical emissions from selected painting micro-samples.

A scheme and a detailed description of the setup is provided in the Supplementary Materials (Figure S2) and here is briefly summarized. In addition to the excitation laser and the image detector, the set-up comprises an optical system, specifically designed for imaging the head of the laser output optical fiber (core diameter Ø = 600 μm) on the sample stage with an uniformly illuminated field of view, independent of the employed objective. The optical system, made of two collecting lenses and of a finite conjugate reflective 15X objective (ReflX™ Objectives, Edmund Optics GmbH, Barrington, NJ, USA), allows the illumination of a field of view of 0.9 mm in diameter with a mean power density on samples of 1 mW/mm^2^. The spatial distribution of the PL signal emitted by the sample and collected by the microscope objective is then detected by the time-gated image intensifier. The system is completed with an excitation filter (FL355-10, Thorlabs Inc., Newton, NJ, USA), a dichroic filter (LPD01-355RU, Semrock, Lake Forest, IL, USA) and a set of nine band pass transmission filters (FKB-VIS-40, Thorlabs Inc.) mounted on a filter wheel in front of the gated camera. The transmission filters allow the detection of the PL emission in selected spectral bands (40 nm FWHM) within the spectral range of 380–870 nm (Figure S3 in Supplementary Materials). The lateral resolution of the device, which is essentially limited by the spatial resolution of the image intensifier (57 lp/mm), has been estimated as 0.9 μm.

The developed TRPL microscope allows the detection and the discrimination between short-lived and long-lived emissions in samples (Figure S4 in Supplementary Materials) on the basis of the following measurement and data analysis protocols:

◾ *Spectral measurements at the nanosecond (ns) and microsecond (μs) timescale*: a sequence of PL gated images at a fixed delay is recorded in different spectral bands. In the present case study, analysis of ns and μs emissions are achieved by employing a gates with a temporal width of W = 10 ns synchronous with laser pulse (delay D = 0 ns) and a gate with a temporal width of W = 10 μs set at a delay D = 0.2 μs after the pulsed excitation, respectively.
◾ *Spectral data analysis:* following correction for the detector efficiency, it is possible to reconstruct the PL spectrum in selected regions of interest (ROIs) of the analyzed sample. In this reconstruction procedure, for the sake of simplicity, each bandpass filter is modeled as a Dirac delta function centered at the filter central wavelength and the spectral transmission of filters are accounted for in the overall spectral detection efficiency (Figure S3 in Supplementary Materials). ROIs are selected on the basis of intensity thresholds on a selected spectral image. Following selection, the PL spectrum in each ROI is shown as the mean of intensity values within the ROI with error bars reporting the ROI standard deviation.
◾ *Decay kinetic measurements at the ns and μs timescale:* a sequence of PL gated images at a fixed spectral band is recorded at different delays with respect to laser pulses. For this work, analysis of ns emission decay kinetics is achieved by employing a gate with a temporal width of W = 10 ns and temporally sampling the emission decay kinetic from 0 to 60 ns. Emission decay kinetics for the µs timescale are analyzed by employing a gate with temporal width of W = 1 μs and temporally sampling the emission decay kinetic from 0.1 to 10 μs.
◾ *Decay kinetic analysis:* it is possible to reconstruct the emission decay kinetic in each point of the analyzed sample. A qualitative estimate of lifetime heterogeneities in the field of view is first provided by the lifetime map, calculated by fitting the data with a simple mono-exponential decay model on a pixel-by-pixel basis [28]. Following this, the emission decay kinetics of selected areas (ROIs) of the specimen are extracted and analyzed through non-linear fitting of a multi-exponential decay model with a maximum of three components. In the employed decay model, the intensifier gate has been considered as a rectangular function of width W, and the temporal width of laser pulses (~1 ns) has been neglected [31]. As for spectral analysis, ROIs are selected on the basis of intensity thresholds. Following selection, the PL decay kinetic in each ROI is shown as the mean of intensity values within the ROI with error bars reporting the ROI standard deviation.

### 2.3. Optical Microscopy and Scanning Electron Microscopy with Energy-Dispersive X-ray Spectroscopy (SEM-EDX)

A commercial optical microscope (Leica DM RE, Easley, SC, USA), equipped with a color digital camera (NIKON D750, Tokyo, Japan), was employed for sample observation in dark-field illumination and in epifluorescence configuration. In the latter case, images were obtained by exciting fluorescence with the 365 nm line of a mercury lamp, while detection was performed in the visible spectral range by employing proper dichroic (T365lpxt, Chroma Technology Corporation, Bellows Falls, VT, USA) and transmission (FELH0400, Thorlabs Inc., Newton, NJ, USA) filters.

A Scanning Electron Microscope (SEM) (Philips XL30 CP), coupled with Energy-Dispersive X-ray spectroscopy (EDX) (Oxford Instruments AZTEC, Oxford, UK), was employed for detecting the spatial distribution of the elemental composition in selected areas of samples. For the purpose, samples were analyzed under high vacuum conditions using a using an accelerating voltage of 20 kV.

## 3. Results

### 3.1. Sample 2105

**Figure 1:**
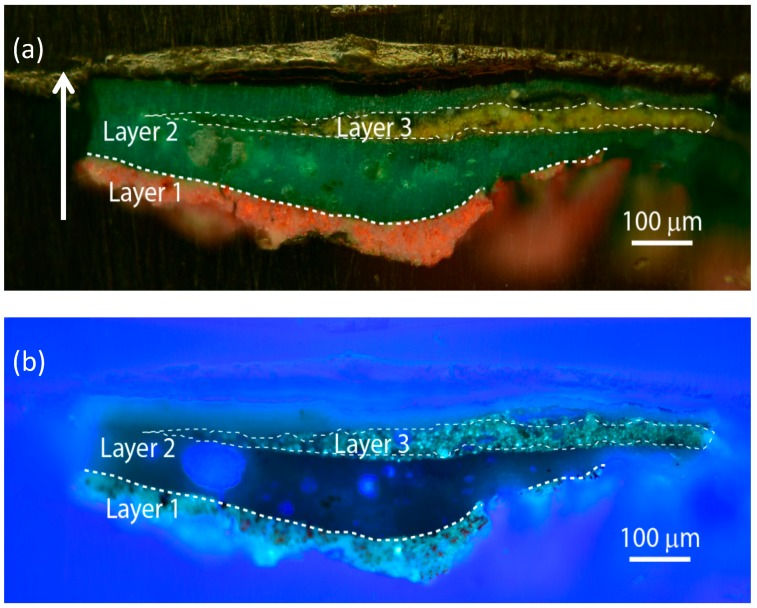
Images of the stratigraphic microsample 2105 taken with the benchtop optical microscope in dark-field illumination (**a**) and epifluorescence configuration (**b**). Borders between painted layers within the sample are highlighted with white dashed lines. The stratigraphic sample is shown in its upright position (as indicated by the white arrow) with the inner layers of the painting being displayed at the bottom of the images.

Sample 2105 (Figure 1), taken from a green painted area of the painting (Figure S1 in Supplementary Materials), showed a complex stratigraphy made of a light red layer (layer 1) below a much thicker green layer (layer 2), with a further yellow layer (layer 3) embedded in the latter. Layers 1 and 3 showed detectable PL signals, whereas no emissions were detected in other parts of the sample. In the following, PL data are reported for luminescent layers only.

**Figure 2:**
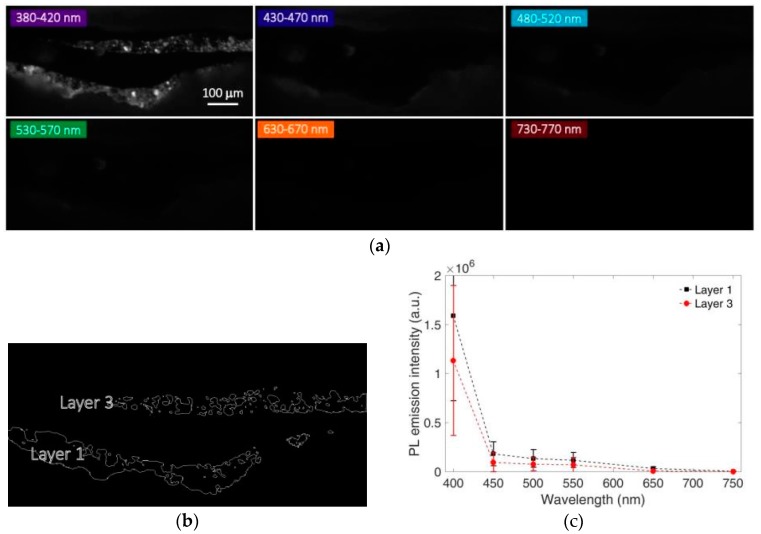
Spectral features of the nanosecond photoluminescence (PL) emission of sample 2105. (**a**) Spatial distribution of the nanosecond PL emission of sample 2105 (delay = 0 ns, gate width = 10 ns) in different spectral bands; (**b**) Regions of interest (ROIs) corresponding to layer 1 and layer 3 of the microsample, selected on the basis of the intensity thresholding of the spectral PL image in the band 380–420 nm; (**c**) Reconstructed mean time-gated PL spectra at the nanosecond timescale detected in ROIs corresponding to layers 1 and 3, with error bars reporting the standard deviation within each ROI.

**Figure 3:**
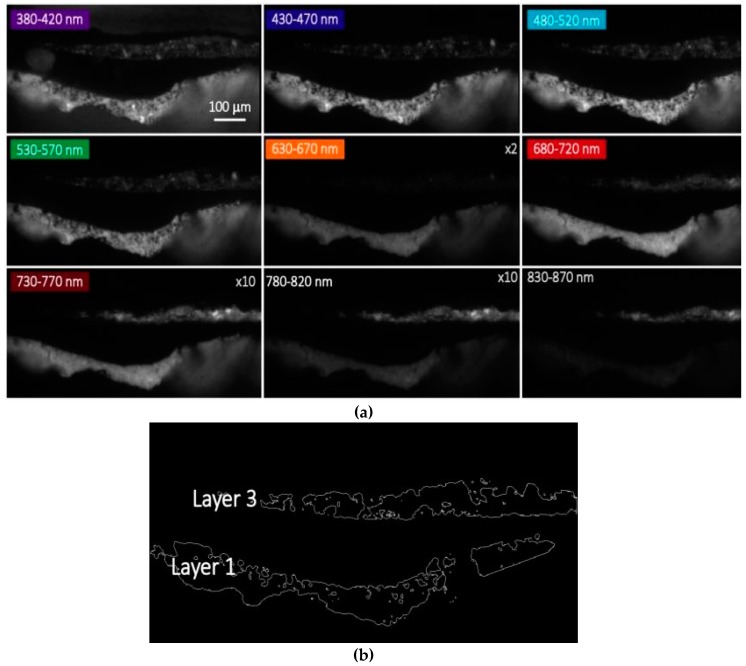

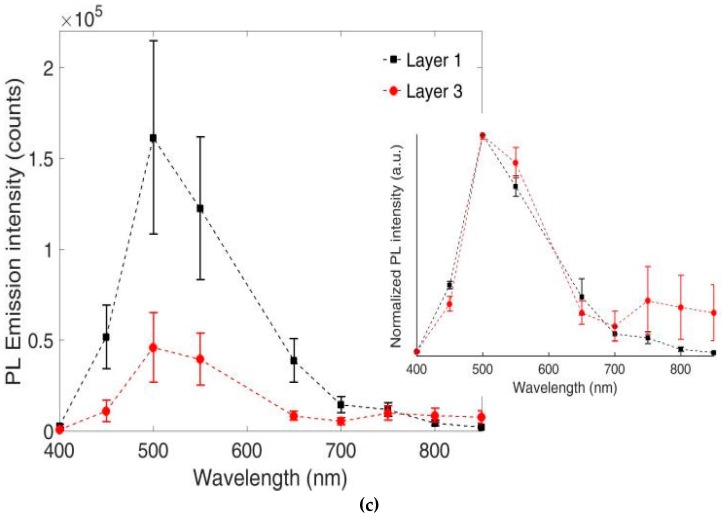
Spectral features of the microsecond PL emissions of sample 2105. (**a**) Spatial distribution of the microsecond PL emission of sample 2105 (delay = 0.2 μs, gate width = 10 μs) in different spectral bands. For better visibility, some images have been amplified by a proper multiplication factor as reported in the figure; (**b**) Regions of interest (ROIs) corresponding to layer 1 and layer 3 of the microsample, selected on the basis of intensity thresholding of the spectral PL image in the band 530–570 nm; (**c**) Reconstructed mean time-gated PL spectra at the microsecond timescales detected in ROIs corresponding to layers 1 and 3, with error bars reporting the standard deviation within each ROI. In the inset, microsecond time-gated PL spectra are shown following normalization at intensity maxima.

Results of multispectral time-gated measurements of the short-lived (nanosecond) and long-lived (microsecond) emissions are displayed in Figure 2 and Figure 3, respectively. In layers 1 and 3 of the sample, we detect a nanosecond intense emission which peaked in the 380–420 nm spectral band (Figure 2), as well as a broader emission occurring at the microsecond timescale and spectrally distributed between 400 nm and 650 nm (Figure 3).

By employing intensity thresholding of gated spectral images we have selected two regions of interest (ROIs) that correspond to the painting layers 1 and 3 of the microsample. In the selected ROIs we have then extracted the gated PL spectra at the nanosecond and microsecond timescales (calculated as mean spectral data in ROIs) that provide an estimate of the mean spectral emissions within layers 1 and 3. These spectra (displayed in the bottom panels of Figure 2 and Figure 3) suggest the use of zinc white (ZnO) as the main pigment employed in the two painted layers on the basis of the well-known PL properties of this semiconductor pigment [7,8,33]. Indeed, ZnO has a bright and narrow emission which is spectrally centered at 380 nm, with an effective lifetime of 1.0 ns, ascribed to direct and exciton-assisted electon-hole recombinations through the bandgap. Moreover, the pigment is characterized by the so-called visible or green emission, spectrally broad with a main center at 520 nm and occurring at the microsecond timescale [33], which originates from a variety of defects within the crystal structure [34].

Interestingly, we observed significant spatial variations of the PL intensity within the two layers in all the blue and green spectral bands (from 400 to 550 nm) (Figure 2 and Figure 3). To the authors’ knowledge, these heterogeneities should be only partially ascribed to variations of ZnO concentration within each layer. Indeed, we suggest that selective reabsorption phenomena [35] of the emitted PL light by the colored (red and yellow) pigment particles play a major role for the observed fluctuations. This hypothesis is confirmed by the fact that PL intensity variations are almost negligible in the spectral bands (from 650 nm to 800 nm), where red and yellow pigments have a low optical absorption.

**Figure 4:**
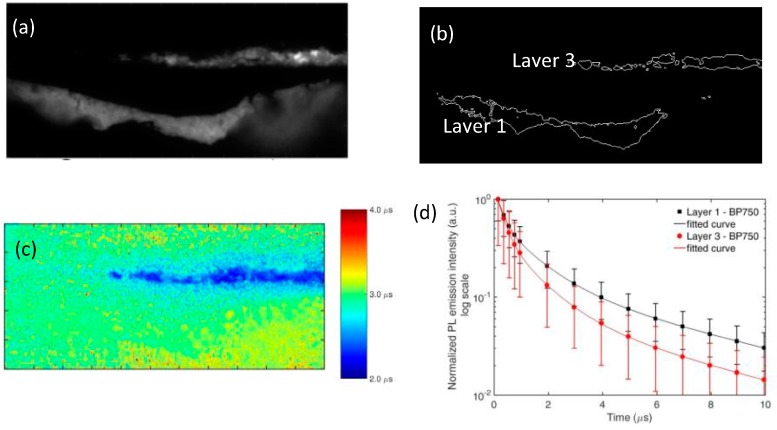
Analysis of the microsecond emission decay kinetics of sample 2105 in the spectral band 730–770 nm. (**a**) Intensity of the PL emission detected at 0.1 µs after pulsed excitation; (**b**) ROIs corresponding to layer 1 and layer 3 of the sample, selected on the basis of intensity thresholding of the PL image shown in (a); (**c**) PL lifetime image reconstructed following mono-exponential fit of the emission decay kinetic in the temporal range from 0.2 to 10 μs; (**d**) Reconstructed decay kinetic of the microsecond PL emission detected in the two ROIs corresponding to layer 1 (black filled squares) and layer 3 (red filled circles). Standard deviation values within each layer (ROI) are reported as error bars. Results of non-linear data fitting on the basis of a tri-exponential decay model are reported as continuous lines (Table S2 in Supplementary Materials).

We further report the presence of a faint, but detectable microsecond PL emission in the near-infrared spectral region (centered around 750 nm) in the localized pigment grains of layer 3 (Figure 3). This occurrence suggests the co-existence of a second luminescent compound in this layer, and lifetime analysis confirms this hypothesis. Indeed, when considering the microsecond PL emission in the spectral band 530–570 nm (where zinc white is the main emitter), we detect similar emission decay kinetics in the two layers of the sample (Figure S7 in Supplementary Materials). Lifetime analysis in the spectral band 730–770 nm (Figure 4) highlights the differences in chemical composition between the two layers, with the PL signal detected in layer 3 dumping more rapidly than the one from layer 1.

**Figure 5:**
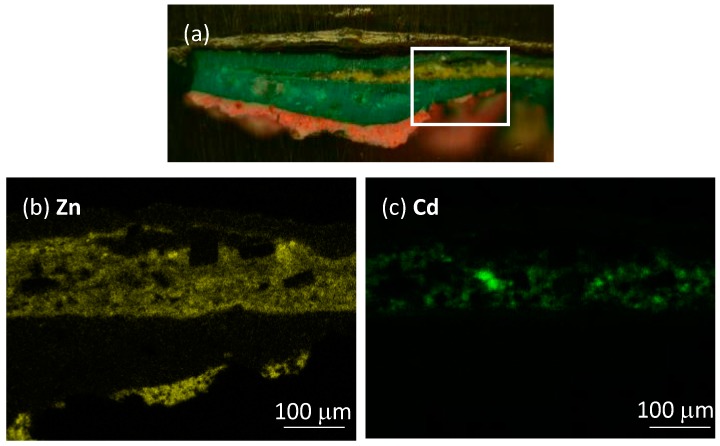
Results of SEM-EDX analysis of microsample 2105 shown as: (**a**) the optical image of the microsample with the white rectangle highlighting the area analyzed by SEM-EDX; elemental maps of Zn (**b**) and Cd (**c**).

SEM-EDX analysis (available as elemental maps in Figure 5 and EDX spectra in Figure S9) performed on a selected area of the micro-sample revealed the presence of Zn, O, Ba, S and Hg as the main elements of layer 1. On the basis of this elemental composition, we confirmed the presence of zinc white mixed with barium white (BaSO_4_) (probably employed as an extender), and with vermillion (HgS) employed to achieve the desired reddish hue of the paint. In layer 3, beside the distinctive elements of zinc white and barium white pigments, we underline the presence of cadmium, hypothesized as cadmium yellow (Zn*_x_*Cd_1−*x*_S) in the layer. Further considerations of this hypothesis will be reported in the Discussion section.

### 3.2. Sample 2107

**Figure 6:**
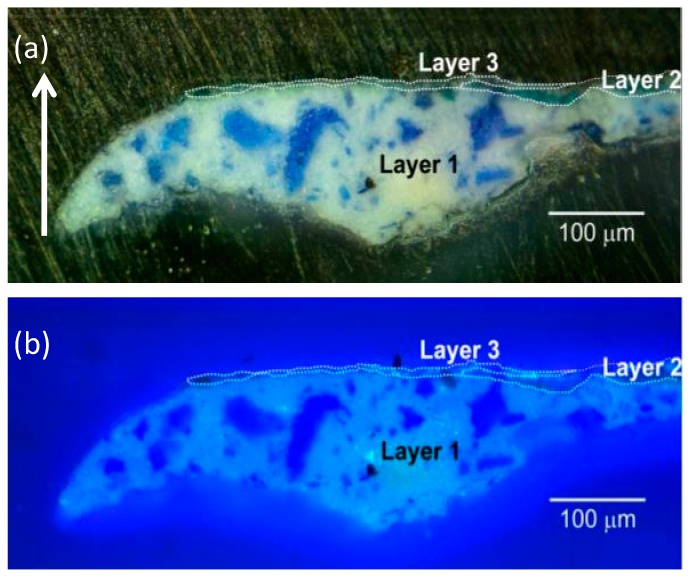
Images of the stratigraphic microsample 2107 taken with the benchtop optical microscope in dark-field illumination (**a**) and epifluorescence configuration (**b**). Borders between painted layers within the sample are highlighted with white dashed lines. The stratigraphic sample is shown in its upright position (with top indicated by the white arrow).

A complex and heterogeneous stratigraphy characterizes the spatial features of sample 2107 (Figure 6), taken from a light blue painted area of the painting (Figure S1 in Supplementary Materials). From bottom to top, we observed the presence of a thick bottom white layer embedding bluish pigment grains (layer 1), a thin green layer (layer 2) and an extremely thin white layer (layer 3).

**Figure 7:**
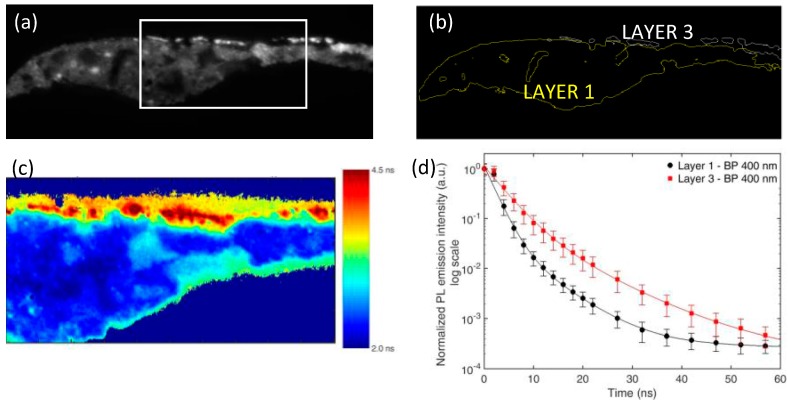
Analysis of the nanosecond emission decay kinetics of sample 2107 in the spectral band 380–420 nm. (**a**) Nanosecond PL gated image detected in the spectral band 380–420 nm; (**b**) ROIs corresponding to layer 1 and layer 3 of sample, selected on the basis of proper intensity thresholding of the PL image shown in (a); (**c**) Lifetime image of the nanosecond PL emission reconstructed on the basis of mono-exponential data fitting in the temporal interval from 0 to 10 ns. For better visibility, results are shown only for the white rectangular area depicted in (a); (**d**) Reconstructed decay kinetic of the nanosecond PL emission detected in ROIs corresponding to layer 1 (black filled circles) and layer 3 (red filled squares). Standard deviation values within each layer are reported as error bars. Results of non-linear data fitting on the basis of a tri-exponential decay model are reported as continuous lines (Table S3 in Supplementary Materials).

Layers 1 and 3 showed detectable, but different PL emissions, suggesting the presence of different luminescent materials (Figure 7 and Figures S10 and S11 in Supplementary Materials). The thin green layer did not show any emissions. In detail, in layer 1 we detected a nanosecond emission with maximum intensity in the 380–420 nm spectral band and a microsecond emission spanning the visible spectral range from 450 nm to 650 nm. As in sample 2105, these PL features let us hypothesize the use of zinc white as the main white pigment of the layer, whereas no indications on the composition of the bluish pigment grains can be given, due to the lack of PL emission.

Conversely, in layer 3 we detected a very bright PL emission in the 380–420 nm spectral band, occurring at both the nanosecond and microsecond timescales, whereas we did not register the microsecond-visible emission characteristic of the zinc white pigment. Analysis of the emission lifetime allowed us to confirm the different nature of the luminescent materials used in the two layers: in particular, in relation to the nanosecond emission decay kinetic in the 380–420 nm spectral band, we detected a substantial longer emission lifetime in layer 3 than in layer 1 (Figure 7).

**Figure 8:**
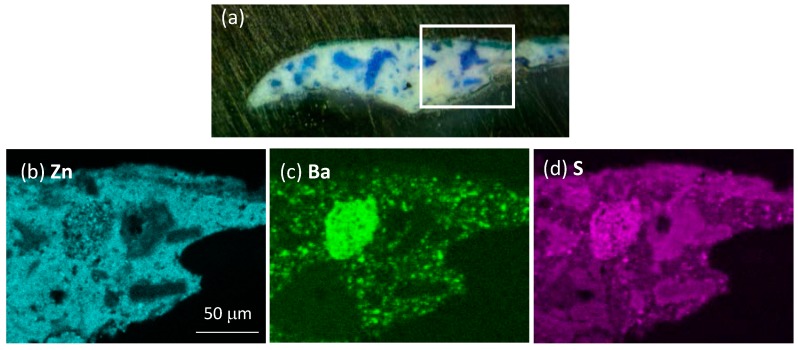
SEM-EDX analysis of sample 2107: (**a**) optical image of the microsample with the white rectangle highlighting the area analyzed by SEM-EDX; (**b**–**d**) spatial distribution of Zn, Ba and S.

Data from SEM-EDX analysis confirm the different nature of the pigments employed in the sample (Figure 8 and Figure S12). Indeed, in layer 1 the presence of Zn, O, Ba and S indicates the use of zinc white as the main white pigment, with barium white used as an extender, in a similar way to that reported for sample 2105. Moreover, the detection of Al and Co in the blue grains (data not reported) suggests the use of the cobalt blue pigment (CoO·Al_2_O_3_). Conversely, in layer 3, we detected Zn and S, but no Ba, thus pointing to the use of zinc sulphide, another white pigment, as the main white pigment of the layer.

## 4. Discussion

For the analysis of painted layers made of luminescent semiconductor pigments, the capabilities of discriminating among spectral and timing PL properties provides key information which is useful for understanding composition. In Table 1 results are summarized in terms of pigment identification provided by the combination of TRPL microscopy and SEM-EDX analysis. 

On the basis of PL data, in two painted layers we have clearly evidenced the presence of the well-known luminescent zinc white pigment. Conversely, the interpretation of the PL signal detected in layer 3 of sample 2105 and sample 2107 require further considerations, which are reported below.

In the yellow layer of sample 2105 (identified as a cadmium-based painted layer by SEM-EDX analysis), we have reported the presence of an infrared luminescent emission. The microsecond time-scale of the emission suggests that it is a defect-related emission occurring in a crystal solid. A first hypothesis is the optical recombination from the first deep trap state of the cadmium yellow pigment (Zn*_x_*Cd_1−*x*_S), characterized by a broad emission spectrally centered between 650 and 750 nm, depending on the Zn molar fraction of the crystal pigment [9,11,36]. Nevertheless, in the analyzed paint layer we have not detected the rapid picosecond emission ascribable to direct recombination paths in CdS. Indeed, following the analysis of cadmium yellow paints produced with contemporary synthesis processes, it has been reported that this emission is easily excited by Q-switch laser pulses [11,37], like the ones employed in the present research. This observation supports two possible hypotheses:

(i) The cadmium yellow paint, produced with the imperfect synthesis processes available at the beginning of the 20th century, is characterized by a high density of crystal defects, which can give rise to the formation of high density of states within the forbidden CdS bandgap. As a consequence, electron trapping of excited electrons is favored with respect to direct recombination, giving rise to a strong quenching of the radiative BE emission.
(ii) The cadmium yellow paint has suffered severe chemical degradation that has altered the original CdS pigment into novel reaction products. In this vision, the detected PL could be ascribed to TS optical emission of novel compounds. This hypothesis is supported by the recent research on altered cadmium yellow paints [18,38,39,40], where pigment degradation has been associated with the formation of cadmium carbonates, sulphates, oxalates and hydroxides.

A clear answer to these hypothesis is not straightforward and would require the use of other complementary analytical methods, which could include synchrotron-based micro-X-ray diffraction and FT-IR mapping. However, it is clear that TRPL microscopy poses important questions regarding possible paint degradation.

**Figure 9:**
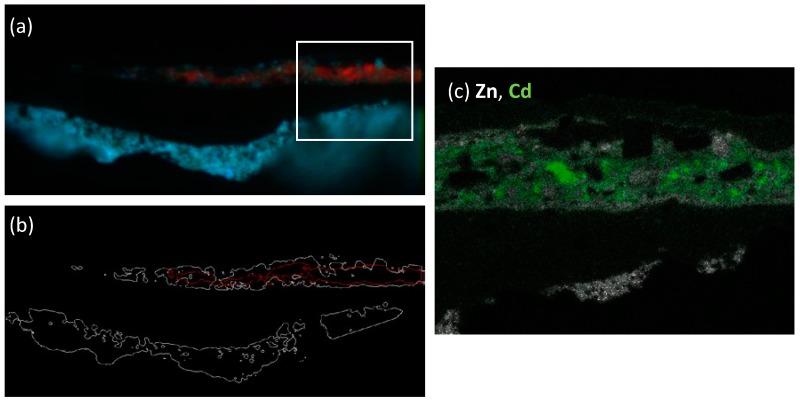
Visualization of the spatial distribution of pigments layer 3 of sample 2105 as highlighted by TRPL microscopy and SEM-EDX analysis: (**a**) false color map created by combining microsecond PL gated images in the spectral bands 430–470 nm (blue), 530–570 nm (green), 830–870 nm (red; amplified in intensity by a factor of 10 for better visibility). The white rectangle highlights the subarea analyzed by SEM-EDX; (**b**) ROIs outlining the spatial distribution of the emission in the green spectral band (530–570 nm) ascribed to zinc white (white contours) and in the infrared spectral band (830–870 nm) ascribed to cadmium yellow (red contours); (**c**) False color map created by combining the SEM-EDX elemental maps of Zn (gray) and Cd (green).

In the analysis of layer 3 of sample 2105, it is of further interest to note that the two main pictorial components (zinc white and cadmium yellow) are non-homogeneosly distributed in the layer, with the yellow pigment being localized in a thinner central region only. This issue is demonstrated in Figure 9 by combining the microsecond emission of the sample in the spectral regions where zinc white is the main emitter (430–470 and 530–570 spectral regions) with the microsecond emission detected in the infrared spectral region (830–870 nm), where cadmium yellow is the main luminescent specie. It is hence clear how results from TRPL microscopy can guide the application of further elemental or molecular spectroscopy analyses in selected areas of samples. Indeed, here similar spatial variations are observed by looking at the spatial distribution of the elemental composition provided by SEM-EDX.

In the analysis of sample 2107, the interpretation of the PL signal detected in layer 3 is not straightforward. Here, we provide some possible interpretations. The use of ZnS as the main white pigment of the layer suggests that the nanosecond and microsecond PL emission detected in the 380–420 (2.9–3.3 eV) spectral band is ascribable to this material. ZnS is an important II–VI semiconductor with a large direct band gap of 3.68 or 3.8 eV, depending on the crystal phase [41]. Today this semiconductor, produced as thin film, quantum dots or nanoparticles, is widely studied as a key material for optoelectronics [42]. PL analysis of materials has elucidated a variety of optical emissions, occurring at different spectral bands and timescales, which are generated by ZnS native defects and uncontrollable impurities depending on the material synthesis process [43]. In particular, an optical emission centered at 396 nm (3.1 eV) has been associated with zinc vacancies in ZnS thin films [44], and this could account for the PL signal detected in the sample.

## 5. Conclusions

Despite being widespread amongst conservators and museum curators, the analysis of the fluorescence emission from samples and artworks is usually employed as a visual and qualitative method for documenting the presence of luminescent compounds. Similar considerations can be reported for the use of fluorescence optical microscopy, which is occasionally applied to stratigraphic samples of paintings to put light on sample heterogeneities. Indeed, conventional optical techniques are limited by the material complexity of artist-painted layers, which comprise heterogeneous mixtures of pigments, organic binders, protective treatments and possible reaction products. For this reason, other analytical microscopies are employed for material identification, such as those based on energy dispersive X-ray, infrared and Raman spectroscopy.

In this work, we have illustrated and proved the effectiveness of TRPL microscopy through the analysis of unique microsamples. The method takes advantage of spectral and lifetime-resolved data and can provide valuable information for the identification of semiconductor pigments in paints. In the specific case study, the analysis has allowed the identification of zinc white and cadmium yellow, and poses specific questions about the degradation of these modern pigments and how luminescence may be modified with chemical changes. In this context, it is important to stress the benefits provided by lifetime analysis with respect to the analysis of PL intensity only. In fact, the independence of the emission lifetime from material concentration allows the detection of the presence of weak PL from pigments even when they are present at very low concentrations.

In conservation science, the method could be extended to the study of other samples and materials, which include ancient photoluminescent pigments, such as Egyptian Blue and lead white. In particular, the sensitivity of the method to the variation of crystal defects in pigments could be addressed to the study of the origin and history of pigments, as has been recently of interest for the lead white pigment [45]. In addition, the analysis of trap state emissions could provide valuable information on painting degradation phenomena that are associated with the migration of ions from pigments (as occurs for the formation of metal carboxylates [15]), and for mechanisms of zinc yellow degradation [39].

## Figures and Tables

**Table 1:** Summary of the main results of TRPL (time-resolved photoluminescence) microscopy and SEM-EDX (scanning electron microscopy-energy dispersive X-ray spectroscopy) analysis and proposed pigment identification.

Sample/Layer	Main PL Emissions (Reported As: Spectral Band of Maximum Intensity/Decay Kinetic Timescale)	Elemental Composition (Main Components)	Proposed Pigment Identification
2105/Layer 1	400 nm/ns timescale (BE); 550 nm/μs timescale (TS)	Zn, O, S, Ba, Hg	zinc white (ZnO); cinnabar (HgS)
2105/Layer 3	400 nm/ns timescale (BE); 550 nm/μs timescale (TS)	Zn, O, S, Ba, Cd, Cr (trace)	zinc white (ZnO); barium white (BaSO_4_); cadmium yellow (Zn_x_Cd_1−x_S)—(altered or unperfectly synthetized)
	750 nm/μs timescale (TS)		
2107/Layer 1	400 nm/ns timescale (BE); 550 nm/μs timescale (TS)	Zn, O, Ba, S, Al, Co	zinc white (ZnO); barium white (BaSO_4_); cobalt blue (CoO·Al_2_O_3_)
2107/Layer3	400 nm/ns timescale (BE or shallow TS); 400 nm/μs timescale (TS)	Zn, S	zinc sulphide (ZnS)

## Supplementary Materials

The following are available online at www.mdpi.com/1996-1944/10/11/1335/s1. Figure S1: Image of the painting *Still Life with Eggplant* by Henri Matisse (Musee des Beaux-Arts, Grenoble, France, 210 × 245 cm^2^ (1911)). The location of microsampling is indicated by numbered black or white squares; Figure S2: Top panel: Scheme of the TRPL-microscopy set-up designed for the present experiment. The black rectangle encloses the optical system specifically designed for achieving a uniform illumination of the sample field of view. Bottom panel: Scheme of the designed optical system with focal lengths and distances between lenses listed in the bottom table; Figure S3: Optical transmission of the employed bandpass filters (colored continuous lines) and estimated relative efficiency of the detection path (dashed black line). The latter has been calculated by modeling each bandpass filter transmission as a Dirac delta function centered at the filter central wavelength and by taking into account the spectral efficiency of the image intensifier input window, of the collecting mirrors and of the bandpass filters; Figure S4: Simplified scheme of the typical PL emission paths in a direct bandgap semiconductor material (with VB and CB referring to the semiconductor valence and conduction band) depicted in terms of energy levels (left panel) and emission decay kinetics (right panel); Figure S5: Spatial distribution of the nanosecond PL emission of sample 2105 (delay = 0 ns, gate width = 10 ns) in the spectral bands 380–420 nm, 430–470 nm, 480–520 nm, 530–570 nm, 630–670 nm, 730–770 nm; Figure S6: Spatial distribution of the microsecond PL emission of sample 2105 (delay = 0.2 μs, gate width = 10 μs) in the spectral bands 380–420 nm, 430–470 nm, 480–520 nm, 530–570 nm, 630–670 nm, 680–720 nm, 730–770 nm, 780–820 nm, 830–870 nm. For better visibility, some images have been amplified by a proper multiplication factor as reported in the figure; Figure S7: Reconstructed decay kinetic of the nanosecond PL emission in the spectral band 380–420 nm detected in layer 1 (black filled squares) and layer 3 (red filled circles) of sample 2105. Standard deviation values within each layer are reported as error bars. Results of non-linear data fitting on the basis of a tri-exponential decay model are reported as continuous lines; Figure S8: Reconstructed decay kinetic of the microsecond PL emission in the spectral bands 530–570 nm (top panel) and 730–770 nm (bottom panel) detected in layer 1 (black filled squares) and layer 3 (red filled circles) of sample 2105. Standard deviation values within each layer are reported as error bars. Results of non-linear data fitting on the basis of a tri-exponential decay model are reported as continuous lines; Figure S9: SEM-EDX analysis of sample 2105: (a) optical image of the microsample with the white rectangle highlighting the area analyzed by SEM-EDX; (b) back-scattered electron map; (c–f) spatial distribution of Zn, Hg, Cd and S; (g) SEM-EDX spectra detected in selected areas (highlighted by white rectangles in (b)) of layer 1 and 3; Figure S10: Spatial distribution of the nanosecond PL emission of sample 2107 (delay = 0 ns, gate width = 10 ns) in the spectral bands 380–420 nm, 430–470 nm; Figure S11: Spatial distribution of the microsecond PL emission of sample 2107 (delay = 0.2 μs, gate width = 10 μs) in the spectral bands 380–420 nm, 430–470 nm, 480–520 nm, 530–570 nm, 630–670 nm, 680–720 nm, 730–770 nm, 780–820 nm. For better visibility, some images have been amplified by a proper multiplication factor as reported in the figure; Figure S12: SEM-EDX analysis of sample 2107: (a) optical image of the microsample with the white rectangle highlighting the area analyzed by SEM-EDX; (b) back-scattered electron map; (c–f) spatial distribution of Zn, S, Cr and Ba; (g) SEM-EDX spectra detected in selected areas (highlighted by white rectangles in (b)) of layer 1 and 3; Table S1: Results of non-linear data fitting of the nanosecond PL emission in the spectral band 380–420 nm detected in layer 1 and layer 3 of sample 2105. The non-linear fit has been performed on the basis of a tri-exponential decay model and in the table results are reported as lifetime and relative amplitude values for each component; Table S2: Results of non-linear data fitting of the microsecond PL emission in the spectral bands 530–570 nm and 730–770 nm detected in layer 1 and layer 3 of sample 2105. The non-linear fit has been performed on the basis of a tri-exponential decay model and in the table results are reported as lifetime and relative amplitude values for each component; Table S3: Results of non-linear data fitting of the nanosecond PL emission in the spectral band 380–420 nm detected in layer 1 and layer 3 of sample 2107. The non-linear fit has been performed on the basis of a tri-exponential decay model and in the table results are reported as lifetime and relative amplitude values for each component.
