# Supplementary material for: Time-Resolved Photoluminescence Microscopy for the Analysis of Semiconductor-Based Paint Layers
Source: Materials (Basel). 2017 Nov 21;10(11):1335. doi: 10.3390/ma10111335 (PMC5706282; doi:10.3390/ma10111335)
Supplement: Supplementary file 1 [file materials-10-01335-s001.pdf]

materials

Supplementary Material

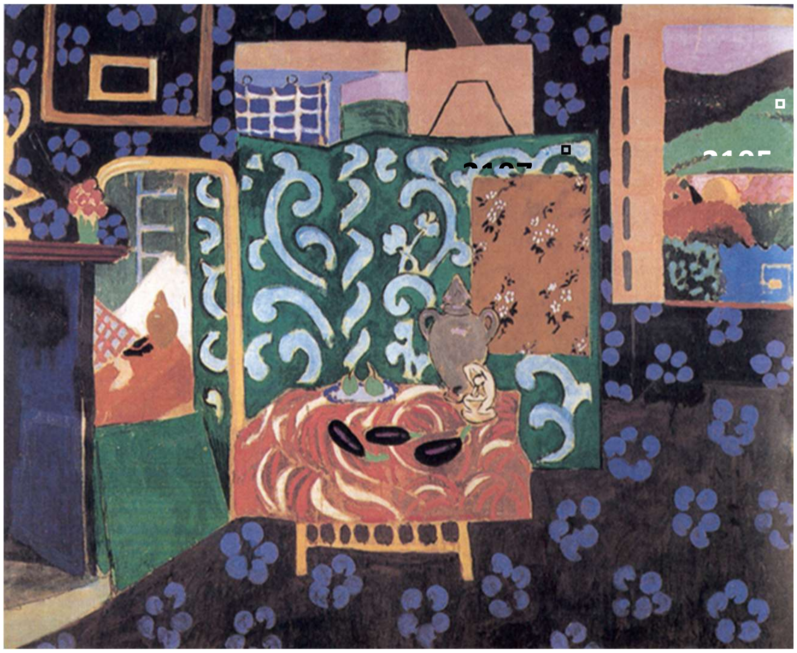

Figure S1. Image of the painting *Still life with eggplant* by Henri Matisse (Musee des Beaux-Arts, Grenoble, France, 210 x 245 cm (1911)). The location of microsampling is indicated by numbered black or white squares.

Details on the developed TRPL microscopy set-up

The system (Figure S2, top panel) is based on a pulsed laser and on a fast-gated intensified camera capable of high-speed gating to capture the emission decay kinetic from samples. Laser excitation is achieved through a Q-switching frequency-tripled diode-pumped Nd:YAG laser (FTSS 355-50 Crylas GmbH, λ=355 nm, pulse energy = 70 µJ, pulse duration = 1.0 ns, repetition rate = 100 Hz). The laser beam, spectrally filtered by an excitation filter (FL355-10 Thorlabs Inc.), is coupled to a 600 µm silica optical fiber and then projected onto the object plan through a proper optical system in order to achieve a uniform excitation (flat top) all over the field of view of the microscope. The optical system (Figure S2, bottom panel) is made of two collecting lenses and of a finite conjugate reflective 15X objective, N.A. = 0.28 (58-421 ReflX™ Objectives, Edmund Optics GmbH). The use of a reflective objective, instead of more conventional refractive objectives, is required to get rid of the background luminescent signal emitted by impurities or doping materials embedded in glasses of on-the-shelf microscope objectives. In fact, whereas this background signal (characterized by microsecond and millisecond emission decay kinetics) does not affect the detection of intense picosecond and nanosecond emissions, it becomes relevant when one deals with the detection of faint microsecond emissions occurring from deep trap states. For achieving sample excitation in epi-fluorescence configuration a dichroic filter (LPD01-355RU, Semrock) is further inserted into the optical system. The whole set-up allows the illumination of a field of view of 0.9 mm in diameter with a power density on sample of 1 mW/mm^2, equivalent to a fluence per pulse of 10 µJ/mm^2. The spatial distribution of the PL signal emitted by the sample is then collected by the reflective microscope objective and detected by the time-gated image intensifier. Spectral sensitivity is is achieved through a set of 9 band pass transmission filters (FKB-VIS-40, Thorlabs Inc.) (Figure S3) mounted on a filter wheel in front of the gated camera. The latter device is made of a gated intensifier (C9546-03, Hamamatsu Photonics) optically coupled (through a relay lens system) to a cooled monochrome camera (Retiga R6, Qimaging). The intensifier unit mounts a GaAs photocathode with spectral

sensitivity from 380 to 850 nm and features an acquisition gate adjustable from 3 ns to continuous mode. A custom-built trigger unit (acting as a constant fraction discriminator) and a precision digital delay generator (DG535, Stanford Research Systems) complete the system, which has a temporal jitter close to 500 ps.

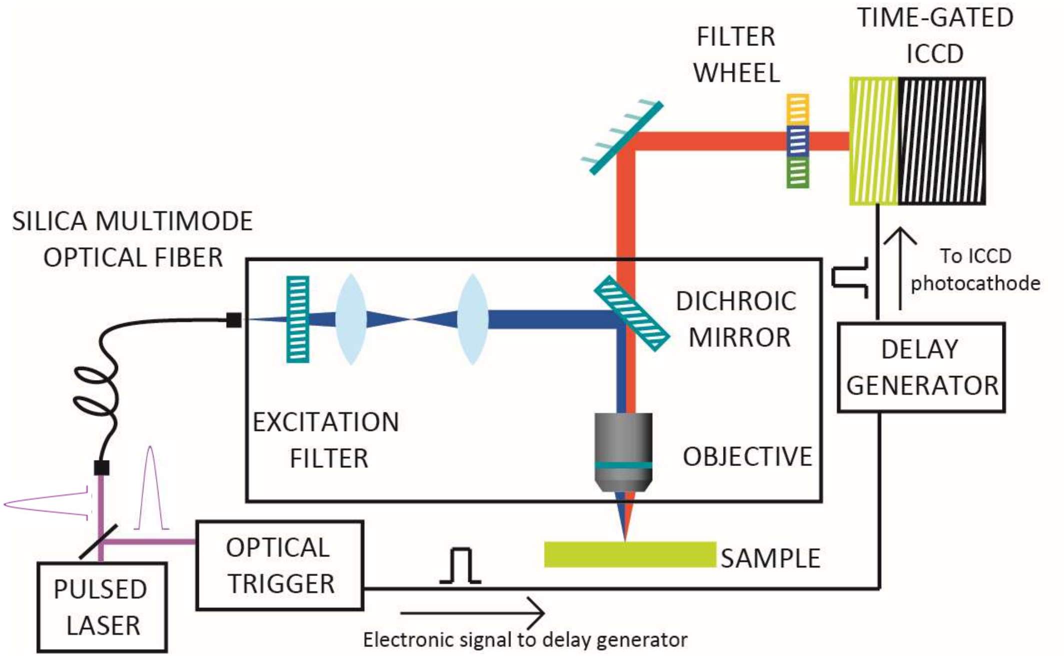

(**top panel**)

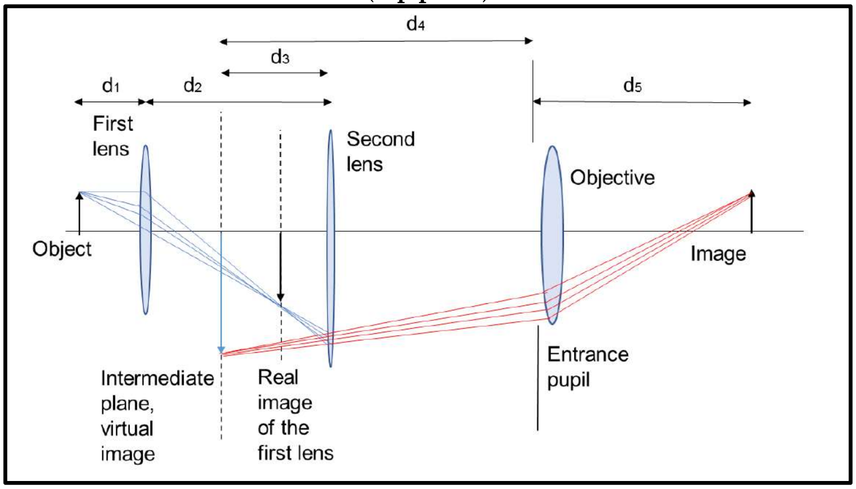

(**bottom panel**)

**Focal length (mm)**	**f_1=15**	**f_2=75**	**f_3=10**		
Distances (mm)	d_1=15.9	d_2=301	d_3=-58.33	d_4=160	d_5=10.7

Figure S2. Top panel. Scheme of the TRPL-microscopy set-up designed for the present experiment. The black rectangle encloses the optical system specifically designed for achieving a uniform illumination of the sample field of view. **Bottom panel.** Scheme of the designed optical system with focal lengths and distances between lenses listed in the bottom table.

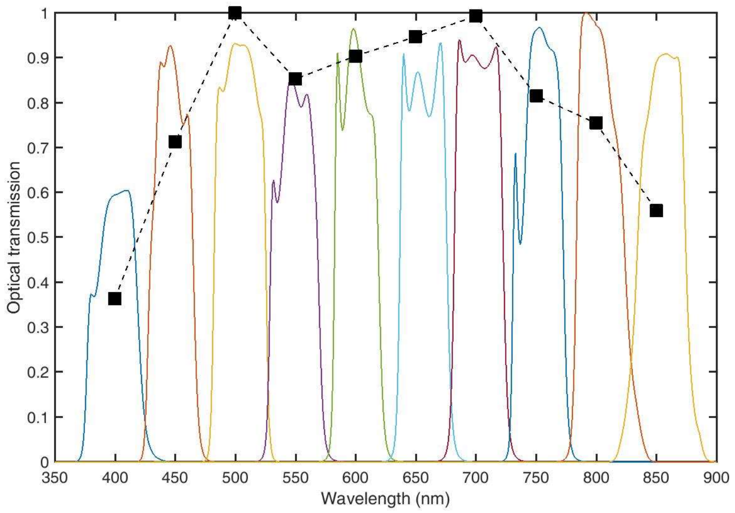

Figure S3. Optical transmission of the employed bandpass filters (colored continuous lines) and estimated relative efficiency of the detection path (dashed black line). The latter has been calculated by modeling each bandpass filter transmission as a Dirac delta function centered at the filter central wavelength and by taking into account the spectral efficiency of the image intensifier input window, of the collecting mirrors and of the bandpass filters.

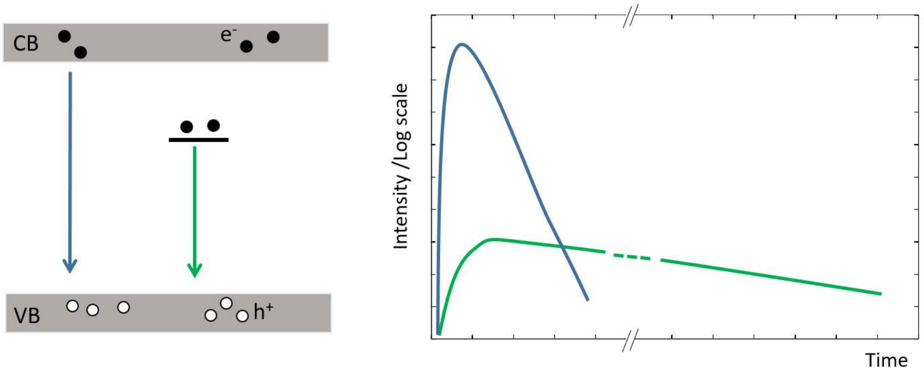

Figure S4 Simplified scheme of the typical PL emission paths in a direct bandgap semiconductor material (with VB and CB referring to the semiconductor valence and conduction band) depicted in terms of energy levels (left panel) and emission decay kinetics (right panel).

Sample 2105 – Spatial distribution of the nanosecond and microsecond PL emissions

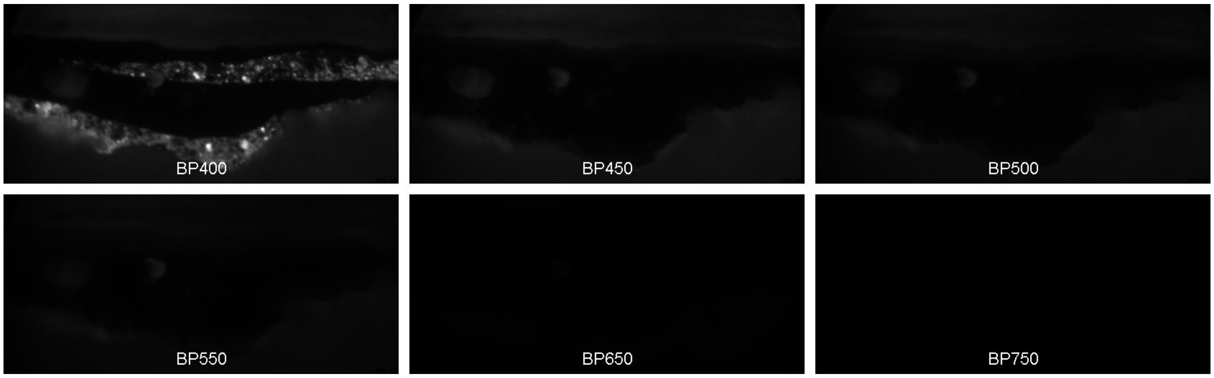

Figure S5. Spatial distribution of the **nanosecond** PL emission of sample 2105 (delay = 0 ns, gate width = 10 ns) in the spectral bands 380-420 nm, 430-470 nm, 480-520 nm, 530-570 nm, 630-670 nm, 730-770 nm.

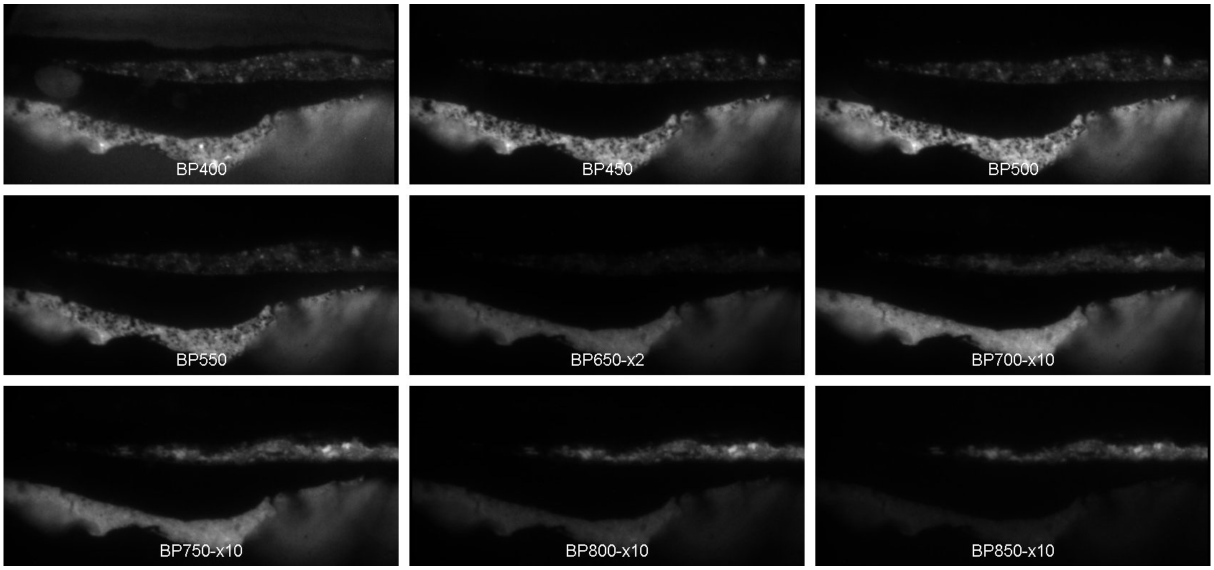

Figure S6. Spatial distribution of the **microsecond** PL emission of sample 2105 (delay = 0.2 μs, gate width = 10 μs) in the spectral bands 380-420 nm, 430-470 nm, 480-520 nm, 530-570 nm, 630-670 nm, 680-720 nm, 730-770 nm, 780-820 nm, 830-870 nm. For better visibility, some images have been amplified by a proper multiplication factor as reported in the figure.

Sample 2105 – Nanosecond and microsecond emission decay kinetic detected in layer 1 and 3 of sample.

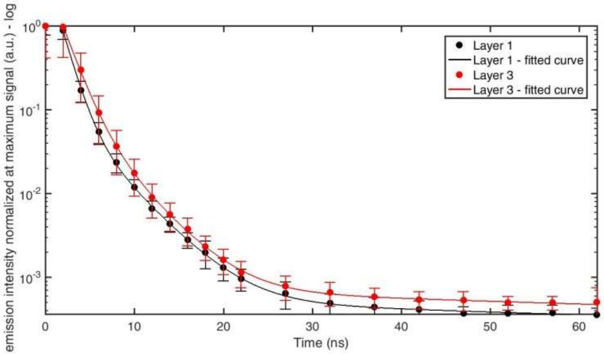

Figure S7. Reconstructed decay kinetic of the nanosecond PL emission in the spectral band 380-420 nm detected in layer 1 (black filled squares) and layer 3 (red filled circles) of sample 2105. Standard deviation values within each layer are reported as errorbars. Results of non-linear data fitting on the basis of a tri-exponential decay model are reported as continuos lines.

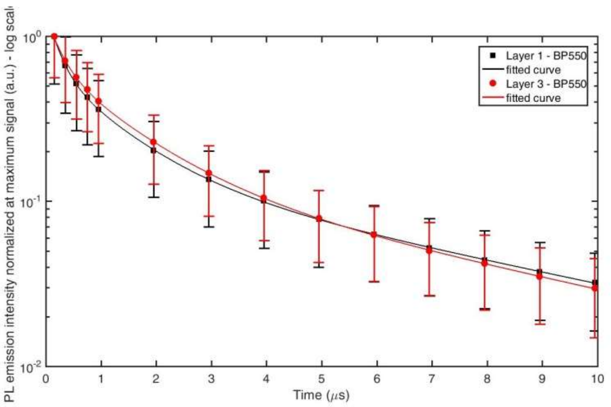

(**top panel**)

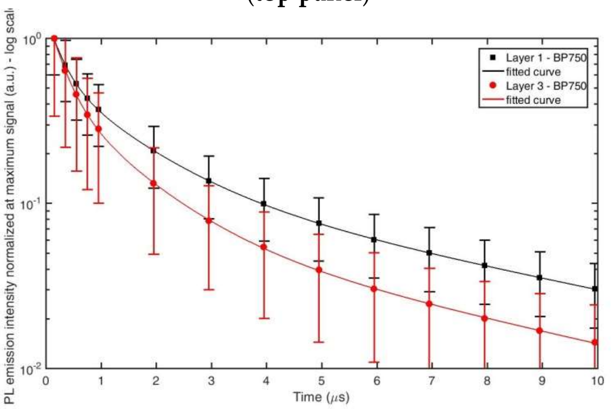

(**bottom panel**)

Figure S8. Reconstructed decay kinetic of the microsecond PL emission in the spectral bands **530-570 nm (top panel)** and **730-770 nm (bottom panel)** detected in layer 1 (black filled squares) and layer 3 (red filled circles) of sample 2105. Standard deviation values within each layer are reported as errorbars. Results of non-linear data fitting on the basis of a tri-exponential decay model are reported as continuos lines.

Table T1: Results of non-linear data fitting of the nanosecond PL emission in the spectral band 380-420 nm detected in layer 1 and layer 3 of sample 2105. The non-linear fit has been performed on the basis of a tri-exponential decay model and in the table results are reported as lifetime and relative amplitude values for each component.

	**τ1 (ns) (A1%)**	**τ2 (ns) (A2%)**	**τ3 (ns) (A3%)**	**R^2**
**Layer 1**	1.1 (99.1%)	3.9 (0.9%)	130.1 (< 0.001%)	0.997
**Layer 3**	1.4 (97.7%)	3.8 (2%)	132.5 (0.003%)	0.997

Table T2: Results of non-linear data fitting of the microsecond PL emission in the spectral bands 530-570 nm and 730-770 nm detected in layer 1 and layer 3 of sample 2105. The non-linear fit has been performed on the basis of a tri-exponential decay model and in the table results are reported as lifetime and relative amplitude values for each component.

	**τ1 (μs) (A1%)**	**τ2 (μs) (A2%)**	**τ3 (μs) (A3%)**	**Effective lifetime (μs)**	**R^2**
**Layer 1 – BP550**	0.21 (80.7%)	1.14 (15.7%)	6.24 (3.5%)	0.57	0.999
**Layer 1 – BP750**	0.25 (78.6%)	1.28 (17.5%)	6.38 (3.9%)	0.68	0.999
**Layer 3 – BP550**	0.25 (73.6%)	1.29 (21.8%)	6.05 (4.0%)	0.74	0.999

**Layer 3 – BP750**	0.27 (83.6%)	1.17 (14.4%)	5.87 (2.0%)	0.52	0.999

Sample 2105 – Results of SEM-EDX analysis

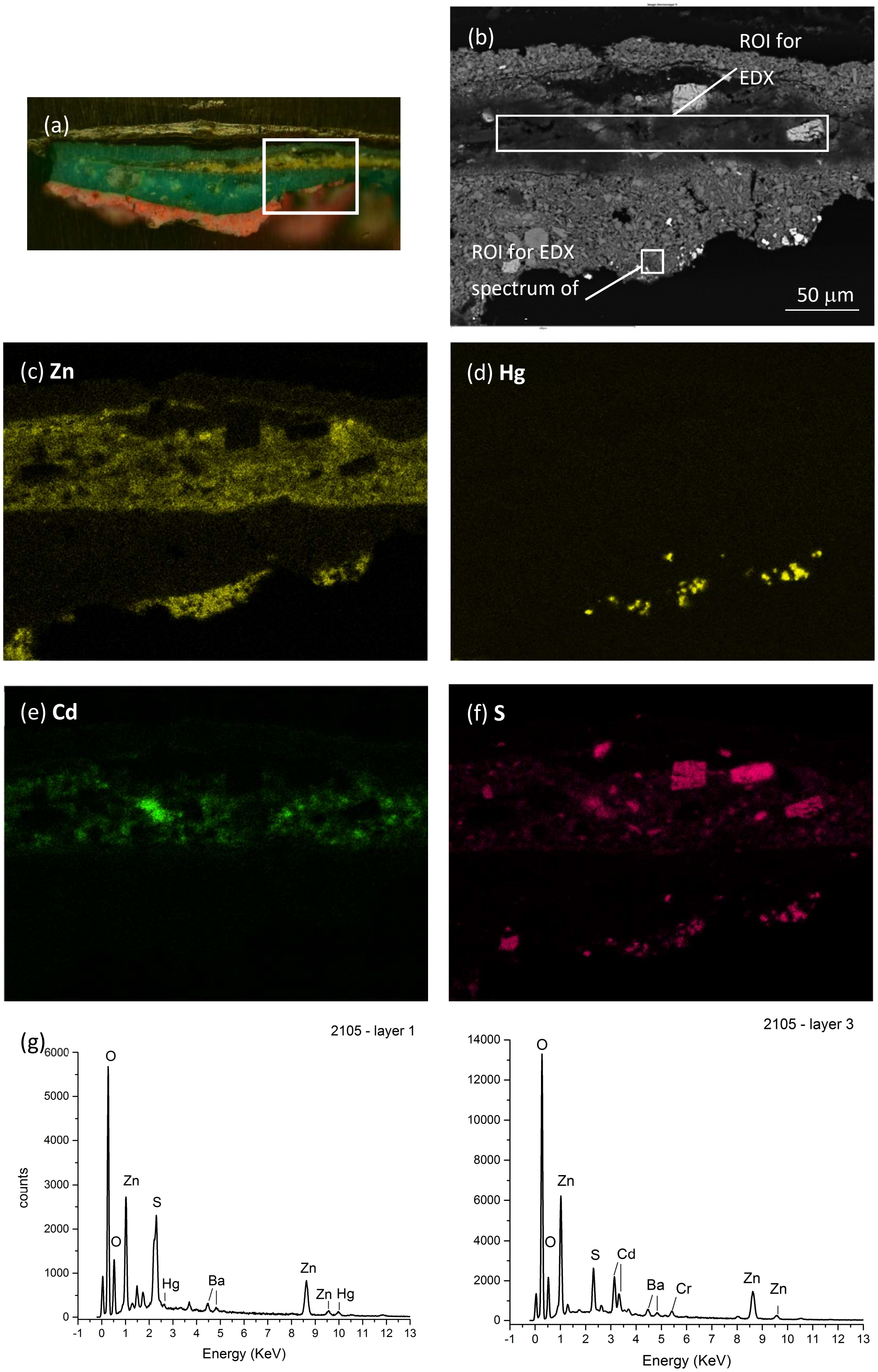

Figure S9. SEM-EDX analysis of sample 2105: (a) optical image of the microsample with the white rectangle highlighting the area analyzed by SEM-EDX; (b) back-scattered electron map; (c-f) spatial distribution of Zn, Hg, Cd and S; (g) SEM-EDX spectra detected in selected areas (highlighted by white rectangles in (b)) of layer 1 and 3.

Sample 2107 – Spatial distribution of the nanosecond and microsecond PL emissions

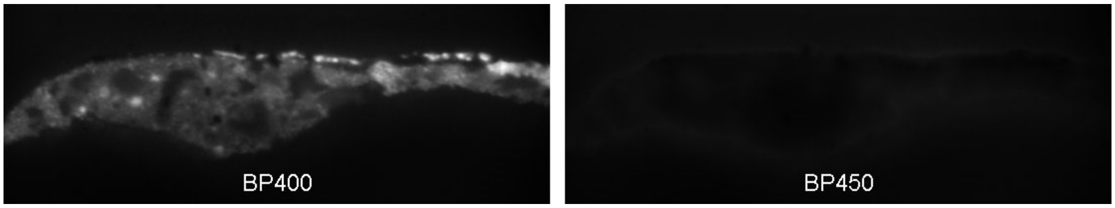

Figure S10. Spatial distribution of the **nanosecond** PL emission of sample 2107 (delay = 0 ns, gate width = 10 ns) in the spectral bands 380-420 nm and 430-470 nm.

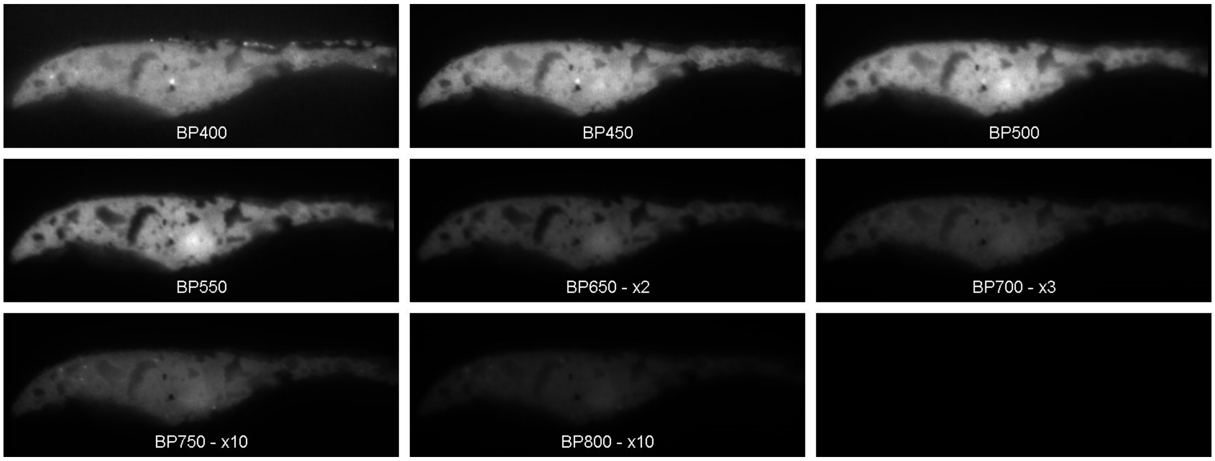

Figure S11: Spatial distribution of the **microsecond** PL emission of sample 2107 (delay = 0.2 μs, gate width = 10 μs) in the spectral bands 380-420 nm, 430-470 nm, 480-520 nm, 530-570 nm, 630-670 nm, 680-720 nm, 730-770 nm, 780-820 nm. For better visibility, some images have been amplified by a proper multiplication factor as reported in the figure.

Table T3: Results of non-linear data fitting of the nanosecond PL emission in the spectral band 380-420 nm detected in layer 1 and layer 3 of sample 2107. The non-linear fit has been performed on the basis of a tri-exponential decay model and in the table results are reported as lifetime and relative amplitude values for each component.

	**τ1 (ns) (A1%)**	**τ2 (ns) (A2%)**	**τ3 (ns) (A3%)**	**R^2**
**Layer 1**	1.9 (98.2%)	6.3 (1.7%)	205.6 (0.01%)	0.971
**Layer 3**	3.4 (93.9%)	8.5 (6.1%)	37.6 (0.05%)	0.982

Sample 2107 – Results of SEM-EDX analysis

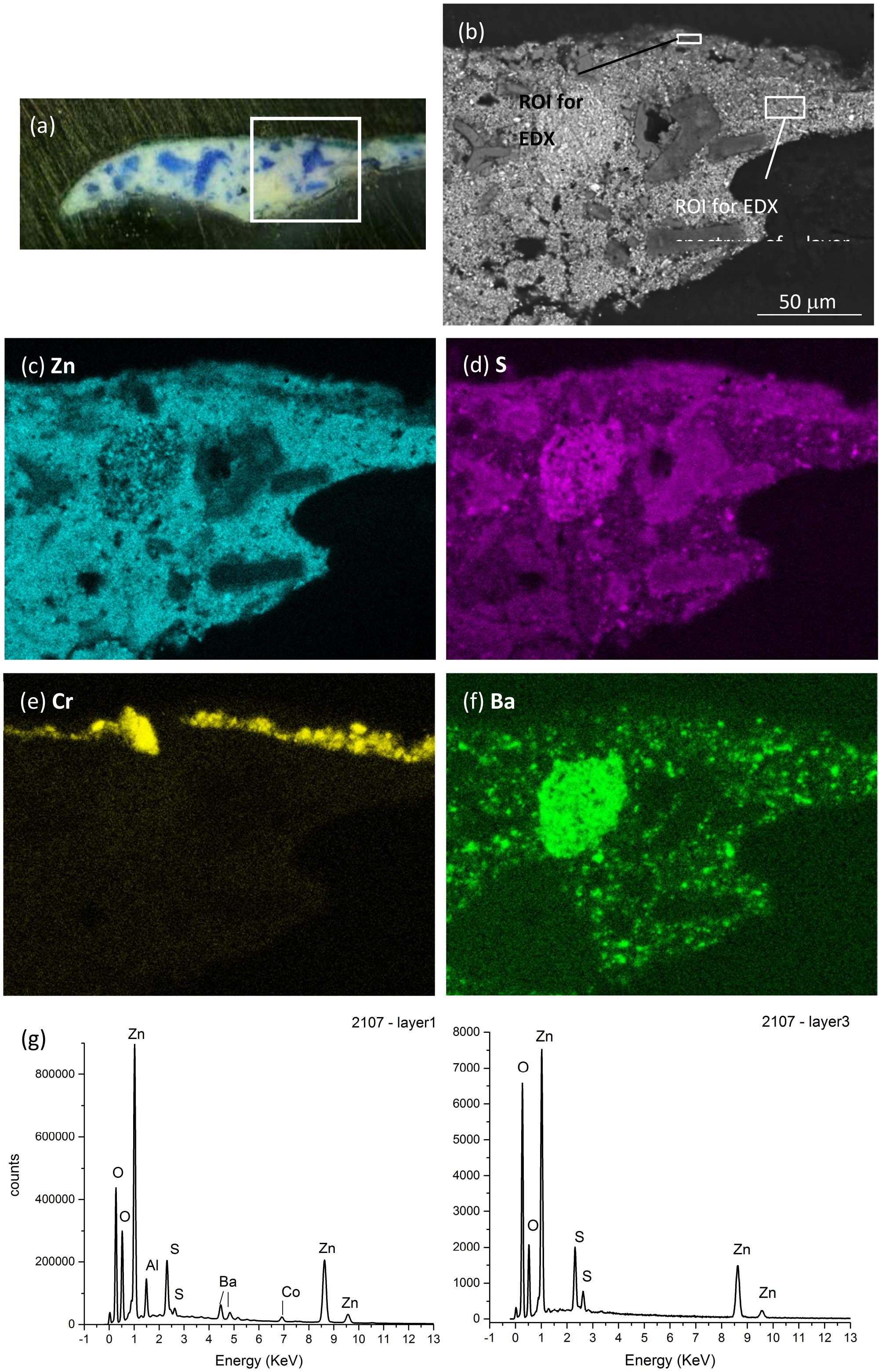

Figure S12. SEM-EDX analysis of sample 2107: (a) optical image of the microsample with the white rectangle highlighting the area analyzed by SEM-EDX; (b) back-scattered electron map; (c-f) spatial distribution of Zn, S, Cr and Ba; (g) SEM-EDX spectra detected in selected areas (highlighted by white rectangles in (b)) of layer 1 and 3**.**
